# Transcranial magnetic stimulation in obsessive-compulsive disorder – a prospective observational study

**DOI:** 10.1192/j.eurpsy.2025.298

**Published:** 2025-08-26

**Authors:** I. Palma, J. Borges, A. L. Ramos, E. Palha, H. Salgado, P. Alves, P. Sousa Martins, R. Moreira

**Affiliations:** 1 Psiquiatria, Unidade Local de Saúde de São João, Porto, Portugal

## Abstract

**Introduction:**

Obsessive-compulsive disorder (OCD) has an estimated prevalence of 1-2% and causes a significative reduction in functionality and quality of life with a high socioeconomic impact.

First and second line treatment includes serotonin reuptake inhibitors, clomipramine, antipsychotics augmentation strategy and cognitive behavioural therapy. It is ineffective in 20-60% of patients whose approach may include transcranial magnetic stimulation (TMS).

**Objectives:**

The aim of this study is to assess the effect of treatment with TMS on obsessive-compulsive, anxious, and depressive symptoms in patients with OCD.

**Methods:**

A prospective observational study was conducted including all patients diagnosed with OCD who underwent TMS in our department since March 2023.

Our protocol targets the dorsolateral prefrontal cortex and includes a total of 25 sessions using the Food and Drug Administration approved parameters (20 Hz, 100% of the leg resting motor threshold, 50 trains of 2s duration, inter-train interval 20s, 2000 pulses per session). Before each session, symptom provocation is performed. TMS was performed using the Cool D-B80 coil and a MagPro stimulator.

Symptoms before and after treatment were assessed using the Yale Brown Obsessive-Compulsive Scale (Y-BOCS), the Hamilton Anxiety Rating Scale (HAM-A) and the Hamilton Depression Rating Scale (HAM-D).

**Results:**

As of 01/09/2024, 21 individuals with OCD completed TMS treatment, 57% male, with a median age of 37 years (interquartile range (IQR) 17). The median duration of illness was 25 years (IQR 15), with 24% of patients having very severe OCD, 52% severe, and 19% moderate—all refractory to psychotherapeutic and pharmacological treatment.

There was a statistically significant reduction in Y-BOCS scores (pre-TMS median score 29 (IQR 6), post-TMS 21 (IQR 13), p=0.003), with 32% of patients achieving a complete response (Y-BOCS reduction ≥35%) and 5% a partial response (Y-BOCS reduction ≥25%). No correlation was found between the change in Y-BOCS scores and other variables such as age, duration of illness, and pre-TMS scores on Y-BOCS, HAM-A, and HAM-D.

Additionally, a statistically significant reduction was observed in HAM-A scores (pre-TMS median 20 (IQR 18), post-TMS 16 (IQR 11), p=0.026) and HAM-D scores (pre-TMS median 19 (IQR 17), post-TMS 15 (IQR 14), p=0.029).

No severe adverse effects were reported.

**Conclusions:**

This study shows significant reductions in Y-BOCS, HAM-A, and HAM-D scores after TMS treatment, with many patients achieving complete or partial response. These findings align with previous research, suggesting TMS is an effective option for treatment-refractory OCD. The absence of severe adverse effects supports its safety.

In conclusion, this study adds to real-world evidence by demonstrating the efficacy and safety of TMS in a clinical setting. Continued data collection is crucial to identify predictors of response.

**Disclosure of Interest:**

None Declared

